# Supplementary material for: Auxin-sensitive Aux/IAA proteins mediate drought tolerance in Arabidopsis by regulating glucosinolate levels
Source: Nat Commun. 2019 Sep 6;10:4021. doi: 10.1038/s41467-019-12002-1 (PMC6731224; doi:10.1038/s41467-019-12002-1)
Supplement: Supplementary file 1 — Supplementary Information [file 41467_2019_12002_MOESM1_ESM.pdf]

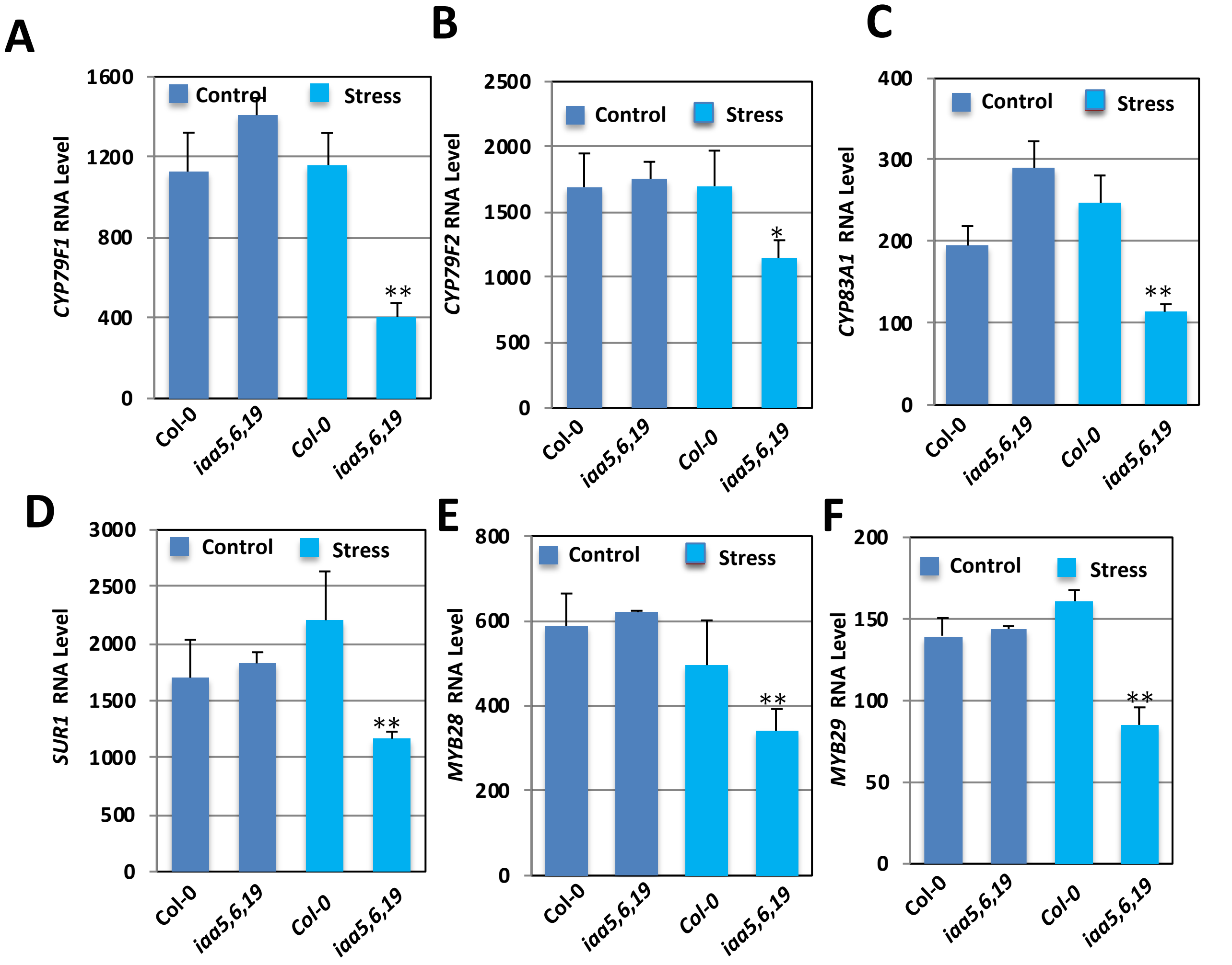

Supplementary Figure 1. **Aliphatic glucosinolate biosynthesis genes are downregulated in the *iaa5,6,19* mutant during dehydration stress.** **(A)** qRT PCR data showing actual RNA values of *CYP79F1*. **(B)** *CYP79F2*, **(C)** *CYP83A1*, **(D)** *SUR1*, **(E)** *MYB28*, **(F)** *MYB29*. For panels differences between the mutant and corresponding Col-0 control are significant at $p < 0.05$ (*) and $p < 0.01$ (**) by two-tailed Student's t test.

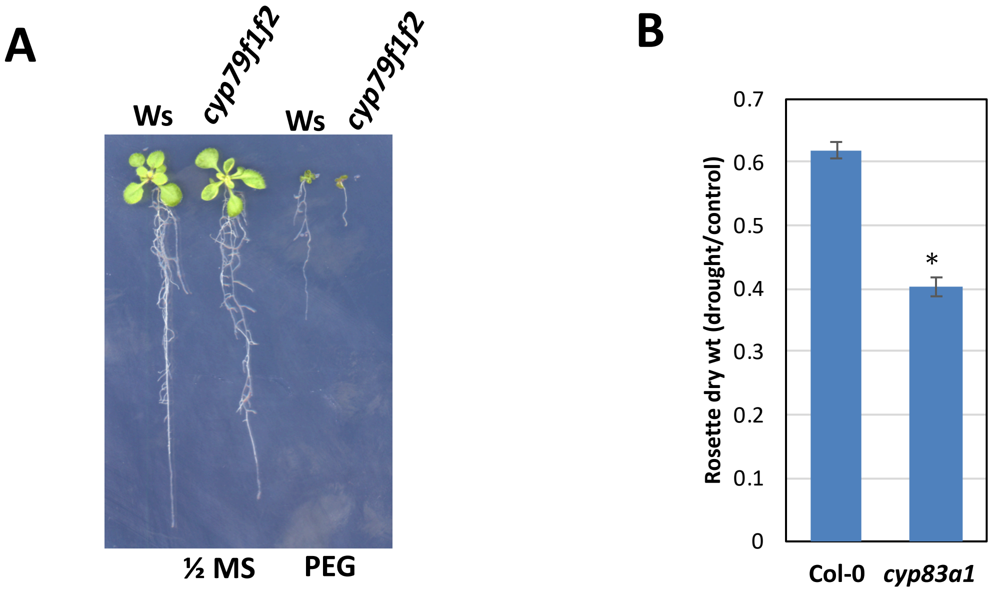

Supplementary Figure 2. Aliphatic glucosinolate biosynthetic mutants are less tolerant of drought or dehydration. (**A)** Representative *cyp79f1f2* seedlings after PEG stress. (**B**) The *cyp83a1* mutant is less drought tolerant than wild type in a water withholding experiment. The differences between mutant and wild type is significant at $p < 0.05$ (*) by two-tailed Student's t test.

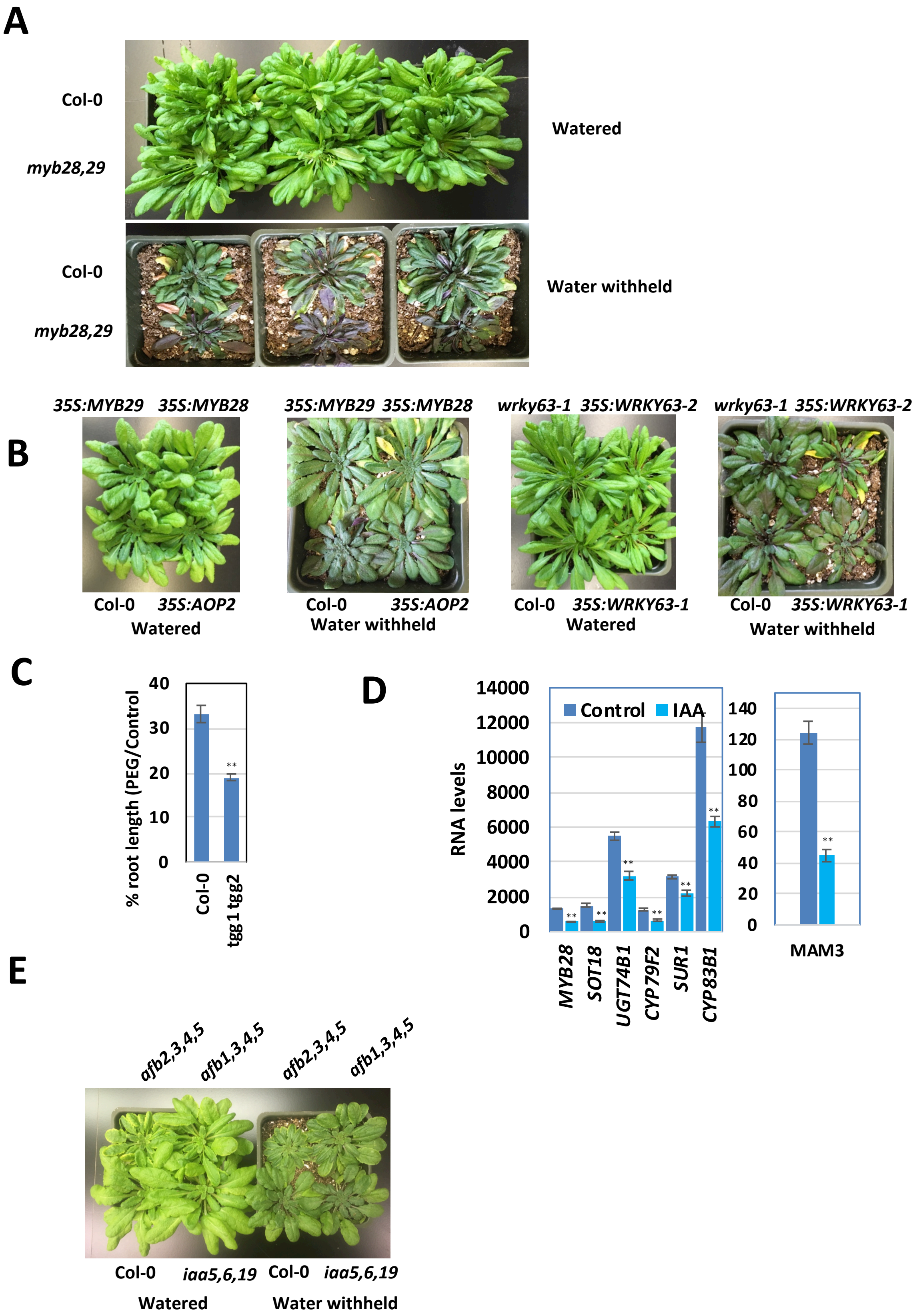

A
Col-0
myb28,29
Watered
Col-0
myb28,29
Water withheld
B
35S:MYB29 35S:MYB28
Col-0 35S:AOP2
Watered
35S:MYB29 35S:MYB28
Col-0 35S:AOP2
Water withheld
wrky63-1 35S:WRKY63-2
Col-0 35S:WRKY63-1
Watered
wrky63-1 35S:WRKY63-2
Col-0 35S:WRKY63-1
Water withheld
C
% root length (PEG/Control
40
30
20
10
0
**
Col-0
tgg1 tgg2
D
RNA levels
14000
12000
10000
8000
6000
4000
2000
0
Control
IAA
**
MYB28
SOT18
UGT74B1
CYP79F2
SUR1
CYP83B1
140
120
100
80
60
40
20
0
MAM3
E
afb2,3,4,5
afb1,3,4,5
afb2,3,4,5
afb1,3,4,5
Col-0
iaa5,6,19
Col-0
iaa5,6,19
Watered
Water withheld

Supplementary Figure 3. Response of *MYB28, MYB29, AOP2* and *WRKY63* mutant and transgenic plants to water withholding. (**A**) The *myb28,29* double mutant is less drought tolerant than the wild type in a water withholding experiment. (**B)** Overexpression of *MYB28*, *MYB29* and *AOP2* results in increased tolerance to water withholding, while *35S:WRKY63* lines are less tolerant in this assay. (**C**) The *tgg1 tgg2* double mutant is sensitive to growth on PEG plates. (**D**) GLS biosynthetic genes are repressed by auxin treatment as shown by qPCR analysis. (**E**) *afb* higher order mutant plants are more drought tolerant than wild type in a water withholding experiment. Differences are significant at $p < 0.05$ (*) and $p < 0.01$ (**) by two-tailed Student's t test.

Supplementary Table 1 -Indolic and aliphatic glucosinolate levels as measured by HPLC in Col-0 and *iaa5,6,19* lines over a period of 3 hours. Each value is the mean +/- SE (nmol/mgFW)

**Indolic GLS**	**Indol-3-ylmethyl glucosinolate**			
	0 min	30 min	60 min	180 min
Col-0	0.0020 +/-0.002	0.0028 +/-0.002	0.0022 +/-0.002	0.0023 +/-0.002
iaa5,6,19	0.0043 +/-0.004	0.0029 +/-0.002	0.0016 +/-0.001	0.0026 +/-0.002
	**4-Methoxyindol-3-ylmethylglucosinolate**			
Col-0	0.0195 +/-0.001	0.0115 +/-0.000	0.0100 +/-0.000	0.0078 +/-0.000
iaa5,6,19	0.0160 +/-0.001	0.0131 +/-0.001	0.0106 +/-0.000	0.0112 +/-0.000
	***N*-methoxy-indol-3-ylmethyl**			
Col-0	0.1021 +/-0.003	0.0833 +/-0.006	0.1025 +/-0.003	0.0820 +/-0.009
iaa5,6,19	0.088 +/-0.005	0.0656 +/-0.005	0.0727 +/-0.006	0.0726 +/-0.003
	**8-methylthio glucosinolate**			
Col-0	0.0919 +/-0.005	0.0815 +/-0.008	0.0874 +/-0.008	0.0748 +/-0.006
iaa5,6,19	0.0891 +/-0.015	0.0630 +/-0.007	0.0640 +/-0.007	0.0623 +/-0.010
**Aliphatic GLS**	**3-Methylsulfinylpropyl glucosinolate**			
Col-0	0.0329 +/-0.004	0.0337 +/-0.001	0.0296 +/-0.006	0.0246 +/-0.005
iaa5,6,19	0.0354 +/-0.003	0.0123 +/-0.003	0.0185 +/-0.005	0.0143 +/-0.004
	**4-Methylsulfinylbutyl glucosinolate**			
Col-0	0.2287 +/-0.032	0.2080 +/-0.030	0.2511 +/-0.018	0.2517 +/-0.021
iaa5,6,19	0.2460 +/-0.030	0.1767 +/-0.016	0.2022 +/-0.018	0.2005 +/-0.025
	**5-Methylsulfinylpentyl glucosinolate**			
Col-0	0.0155 +/-0.000	0.0126 +/-0.000	0.0148 +/-0.001	0.0142 +/-0.001
iaa5,6,19	0.0147 +/-0.001	0.0141 +/-0.001	0.0121 +/-0.001	0.0134 +/-0.002
	**7-Methylsulfinylheptyl glucosinolate**			
Col-0	0.0130 +/-0.000	0.0119 +/-0.000	0.0127 +/-0.001	0.0112 +/-0.001
iaa5,6,19	0.0150 +/-0.000	0.0123 +/-0.000	0.0141 +/-0.001	0.0166 +/-0.001

Supplementary Table 2. Primers used in this stidy

qPCR

**Primer Numb**	**Primer Name**	**Primer Sequence, 5'-3'**	**Primer Purpose**
MS433	qMYB29 F	CGTTGATTGCTTACCGGACT	qPCR of MYB29
MS434	qMYB29 R	AGTGACCCTATAGTGGACCTTTACT	Do
MS435	qCYP79F1 F	CTTGACGTACTGTCGTTTGTTG	qPCR of CYP79F1
MS436	qCYP79F1 R	GCTACTCCGAATGTTTGATCG	Do
MS437	qCYP79F2 F	TGATGTGTTTCGACGCTTTG	qPCR of CYP79F2
MS438	qCYP79F2 R	TATAGCGTTTTCGGGCAATG	Do
MS439	qCYP83A1 F	TGGCAATCGTCTCTCTATCTTTC	qPCR of CYP83A1
MS440	qCYP83A1 R	GACATCATGTGAATTTGCTTCC	Do
MS441	qSUR2 F	CCATCAAATTCACTCACGAAAATGTC	qPCR of CYP8B1/SUR2
MS442	qSUR2 R	AAGGTAAGTCATGGCCCATACCACC	Do
MS443	qSUR1 F	CCGGCAAAGGCAATTCTTACGG	qPCR of SUR1
MS444	qSUR1 R	TCATATAATCAGCAACGGCTCGTC	Do
MS445	qMYB28 F	AGACTGCGATGGACCAACTACC	qPCR of MYB28
MS446	qMYB28 R	TCTCGCTATGACCGACCACTTG	Do
MS451	qWRKY63 F	AACATCGATCACAAGGCTGTGG	qPCR of WRKY63
MS452	qWRKY63 R	TCTTGAGGATGTTAGCGCATCCC	Do

Genotyping and cloning

MS446	qMYB28 R	TCTCGCTATGACCGACCACTTG	Genotyping MYB28 OE
MS457	genoMYB29 1R	TCATATGAAGTTCTTGTCGTCGT	Genotyping MYB29 OE
	35S F	GGGATGACGCACAATCCCACTATC	Genotyping 35Spromoter in MYB28 and MYB29 OE
MS459	cWRKY63_F	CACCTAATATGTTGCTCAACTTTCATAGGAC	For cloning of WRKY63 promoter +genomic DNA without STOP codon into pENTR/D/GW.
MS460	cWRKY63_R	AAACAACATCAGGTCTTCCGA	Do
MS461	sWRKY63_1F	ATTGAAGTTTCCACGCTAT	Sequencing WRKY63
MS462	sWRKY63_2F	CGAGACATGGCAGGTCTTGT	Do
MS463	sWRKY63_3F	CCTTTGGGGTGCATGATAATACG	Do
MS464	sWRKY63_1R	GTTGTCGAGGACCGTCTTGA	Do
MS465	sWRKY63_2R	GCATAGCAGTTTTGGTCTTTTGC	Do
MS466	sWRKY63_3R	ACAACATCAGGTCTTCCGATGA	Do
MS468	cIAA19g_2F	GGACGTGGGAACATGCTTGTAGT	Cloning IAA19pro-IAA19g in pCR8-GW-TOPO
MS425	cIAA19g_R	ACTCAACACTCAAGAAACAAGTAGTGT	Do
MS469	sIAA19_F	TCTCTCATGTGACCGACCAC	Sequencing IAA19
MS470	sIAA19_1R	GCGAGCATCCAGTCTCCATC	Do
MS471	sIAA19_2R	TCAACACTCAAGAAACAAGTAGTGT	Do
MS474	cIAA19_NcoI F	GCCCCATGGATGGAGAAGGAAGGACTCGG	Cloning IAA19 genomic with 3'UTR in pGREENII-0179-GC1p binary
MS475	cIAA19_BsaBI R	GCCGATACGCATCACTCAACACTCAAGAAACAAGTAGTGT	Do
MS476	cDREB2A_NcoI F	GCCCCATGGATGGCAGTTTATGATCAGAGTGGA	Cloning DREB2A CDs in pGREENII-0179-GC1p binary
MS477	cDREB2A_BsaBI R	GCCGATACGCATCTTAGTTCTCCAGATCCAAGTAACTCA	Do
MS478	GC1 F seq primer	TGTGATCTCTATCCAACA	Sequencing DREB2A CDs fusion with GC1 Promoter
MS479	nosT R seq primer	AACGTCATGCATTACATG	Sequencing presence of nosT
MS493	qMAM3_F	CGCCATTCATTGTCATAACG	
MS494	qMAM3_R	CATCACGACCTCTTCAAGC	
MS495	qSOT18_F	CCTCATCTCCACGTTTCCTC	
MS496	qSOT18_R	CATACTCGATCAGGGGCTCT	
MS497	qUGT74B1_F	CGTTTCGTATCCGTGGCTTA	
MS498	qUGT74B1_R	CAGACTCACCATTTTCACAATCT	

ChIP qPCR

MS481	MYB29 q1R	ATAAGCCTGGCACTCGTGTAG	ChIP around W box in MYB29 promoter
MS484	MYB29 q3F	GTCACTAACACTCACTCTTGATG	Do
MS487	MYB28 q2F	GTCAAGAAAGCCATGTTGCG	ChIP around W box in MYB28 promoter
MS488	MYB28 q2R	CACCGTTTCAACCCTAATCAG	
MS489	WRKY63_ChIP_q1F	CTAAATCCTCTCATAGTCTCATC	ChIP around AuxRE in IAA19 promoter
MS490	WRKY63_ChIP_q1R	TCCAGATCCTTTCCCACTTCG	Do
MS491	WRKY63_ChIP_q2F	GAGAAGAAGACAAGTCTACTTTT	Do
MS492	WRKY63_ChIP_q2R	CGAAGAACATCGGCCATGAT	Do
