# Supplementary material for: Auxin-sensitive Aux/IAA proteins mediate drought tolerance in Arabidopsis by regulating glucosinolate levels
Source: Nat Commun. 2019 Sep 6;10:4021. doi: 10.1038/s41467-019-12002-1 (PMC6731224; doi:10.1038/s41467-019-12002-1)
Supplement: Supplementary file 3 — Description of Additional Supplementary Files [file 41467_2019_12002_MOESM3_ESM.pdf]

Description of Additional Supplementary Files

File Name: Supplementary Data 1
Description: Genes differentially regulated in iaa5,5,19 vs Col-0
Down-regulated genes iaa5,6,19 vs Col-0 during stress (FDR < 0.001
